# Supplementary material for: Circulating Mesenchymal Stromal Cells in Patients with Infantile Hemangioma: Evaluation of Their Functional Capacity and Gene Expression Profile
Source: Cells. 2024 Jan 29;13(3):254. doi: 10.3390/cells13030254 (PMC10854919; doi:10.3390/cells13030254)
Supplement: Supplementary file 1 [file cells-13-00254-s001.zip › Table S1.pdf]

Table S1. List of analysed genes

List of genes (n=147) assessed in cMSCs, BM MSCs, ECFCs and HUVECs. Genes (n=48) that resulted differently expressed in MSCs with respect to endothelial cells are shown in boldface/grey cells.

Gene	Refseq ID	Gene	Refseq ID	Gene	Refseq ID
ACE	**NM_000789**	*FGFR1*	NM_023110	*NF1*	NM_000267
ACTA2	NM_001141945	*FGFR3*	NM_0001402	***NOS3***	**NM_000603**
AKT1	**NM_005163**	*FIGF*	NM_004469	*NOTCH1*	NM_017617
ANG	**NM_001097577**	***FLI1***	**NM_002017**	*NRP1*	NM_001244972
ANGPT1	NM_001146	***FLT1***	**NM_001159920**	*NRP2*	NM_003872
ANGPT2	**NM_001118887**	*FLT4*	NM_002020	*OSR1*	NM_145260
ANGPTL4	**NM_001039667**	*FN1*	NM_002026	*PECAM1*	NM_000442
ANPEP	NM_001150	*FUT4*	NM_002033	*PF4*	NM_002619
ATXN1	NM_000332	*GAPDH*	NM_002046	*PGF*	NM_001207012
B2M	NM_004048	*GATA1*	NM_002049	***PLAU***	**NM_001145031**
BAI1	**NM_001702**	***GATA2***	**NM_001145661**	***PLAUR***	**NM_001005376**
BMP2	NM_001200	*GATA3*	NM_001002295	*PLG*	NM_000301
BMP4	NM_001202	*GLI1*	NM_001160045	*PROK2*	NM_021935
CCL2	NM_002982	*HBB*	NM_000518	*PROM1*	NM_001145847
CD33	NM_001082618	***HGF***	**NM_000601**	***PTGS1***	**NM_000962**
CD34	NM_001025109	*HIF1A*	NM_001243084	*RPLP0*	NM_053275
CD38	NM_001775	***HOXB4***	**NM_024015**	*RUNX1*	NM_001001890
CD44	NM_000610	***HPRT1***	**NM_000194**	*S1PR1*	NM_001400
CDH5	NM_001795	*HPSE*	NM_001098540	***SERPINE1***	**NM_000602**
COL18A1	**NM_030582**	*ID1*	NM_002165	*SERPINF1*	NM_002615
COL4A3	NM_000091	*IFNG*	NM_000619	*SMAD2*	NM_001003652
CTGF	**NM_001901**	*IGF1*	NM_000618	*SMAD3*	NM_001145102
CTNNB1	NM_001904	*IKAROS*	NM_001220771	***SPHK1***	**NM_001142601**
CXCL1	NM_001511	***IL1B***	**NM_000576**	*SPI1*	NM_001080547
CXCL10	NM_001565	*IL6*	NM_000600	***SPINT2***	**NM_001166103**
CXCL12	NM_001033886	***IL8***	**NM_000584**	*TAL1*	NM_003189
CXCL12_3	**NM_001178134**	*ITGAV*	NM_001144999	*TEK*	NM_000459
CXCL5	**NM_002994**	*ITGB3*	NM_000212	***TFPI***	**NM_006287**
CXCL6	**NM_002993**	***JAG1***	**NM_000214**	*TFPI2*	NM_006528
CXCL9	NM_002416	***JAK2***	**NM_004972**	***TGFA***	**NM_001099691**
CXCR4-1	NM_003467	***KDR***	**NM_002253**	*TGFB1*	NM_000660
CXCR4-2	NM_001008540	*KRAS*	NM_004985	*TGFB2*	NM_001135599
CXCR4-TOT	-	***LAMC2***	**NM_005562**	*TGFBR1*	NM_004612
DES	NM_001927	*LECT1*	NM_001011705	*TGM2*	NM_004613
EDN1	**NM_001168319**	*LEP*	NM_000230	*THBS1*	NM_003246
EFNA1	**NM_182685**	***LMO2***	**NM_001142315**	*THBS2*	NM_003247
EFNB2	**NM_004093**	*LOX*	NM_001178102	*TIE1*	NM_005424
EGF	**NM_001178130**	*LOXL1*	NM_005576	*TIMP1*	NM_003254
EGFR	NM_005228	*LOXL2*	NM_002318	***TIMP2***	**NM_003255**
ENG	NM_000118	*LOXL3*	NM_032603	*TIMP3*	NM_000362
EPAS1	NM_001430	*LOXL4*	NM_032211	***TNF***	**NM_000594**
EPHA2	**NM_004431**	*MDK*	NM_001012333	***TYMP***	**NM_001113755**
EPHB4	NM_004444	***MLL***	**NM_001197104**	***UBC***	**NM_021009**
ERBB2	NM_004448	*MMP1*	NM_001145938	*VEGFA*	NM_001025366

ESM1	**NM_007036**	*MMP14*	NM_004995	*VEGFB*	NM_001243733
ETS1	NM_001143820	*MMP2*	NM_004530	***VEGFC***	**NM_005429**
F3	**NM_001993**	*MMP9*	NM_004994	*VWF*	NM_000552
FGF1	NM_000800	***MYB***	**NM_001130172**	*WNT1*	NM_005430
FGF2	NM_002006	*MYCN*	NM_005378	***YWHAZ***	**NM_001135699**
