# Supplementary figures and images for: Circulating Mesenchymal Stromal Cells in Patients with Infantile Hemangioma: Evaluation of Their Functional Capacity and Gene Expression Profile
Source: Cells. 2024 Jan 29;13(3):254. doi: 10.3390/cells13030254 (PMC10854919; doi:10.3390/cells13030254)

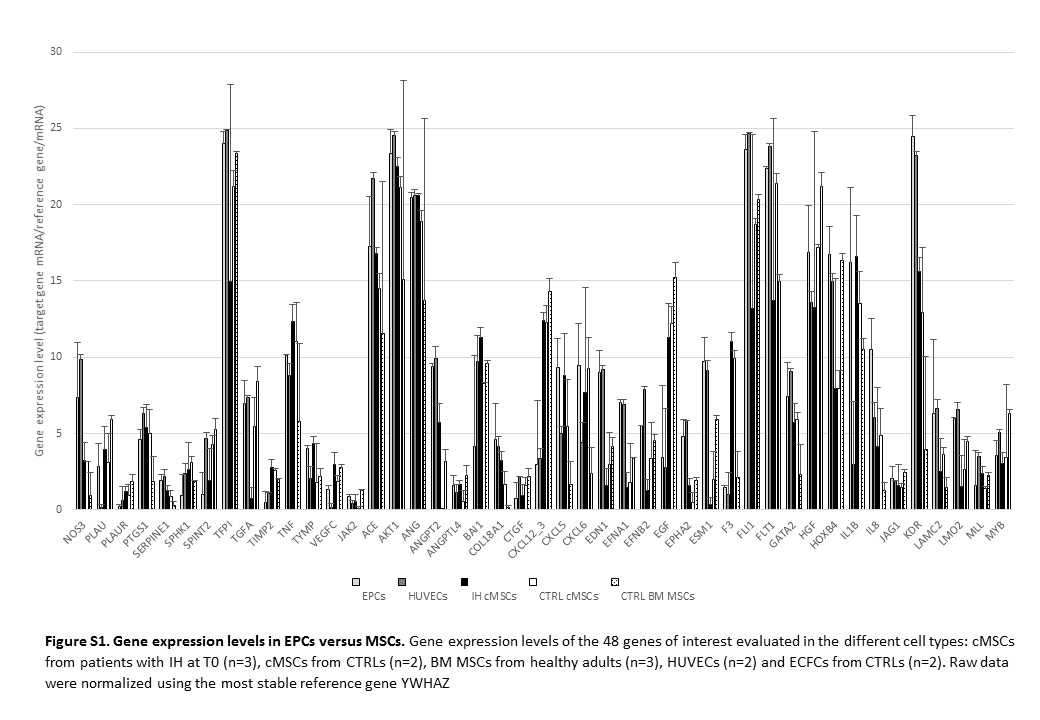

Supplement: Supplementary file 1 [file cells-13-00254-s001.zip › Figure S1.tif]

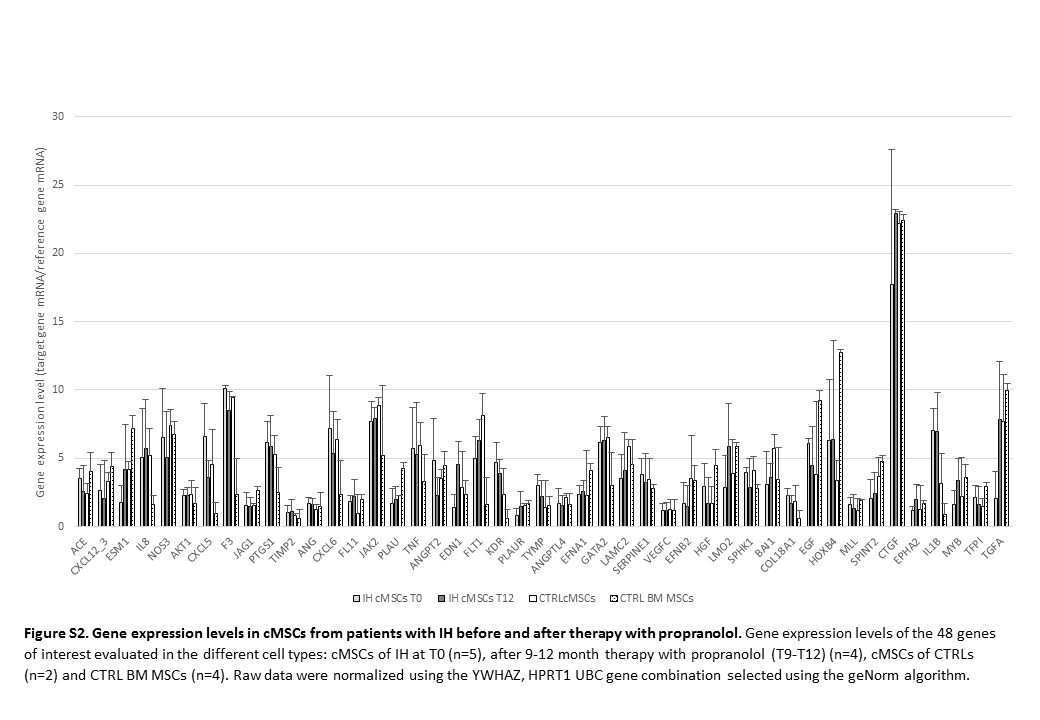

Supplement: Supplementary file 1 [file cells-13-00254-s001.zip › Figure S2.tif]
